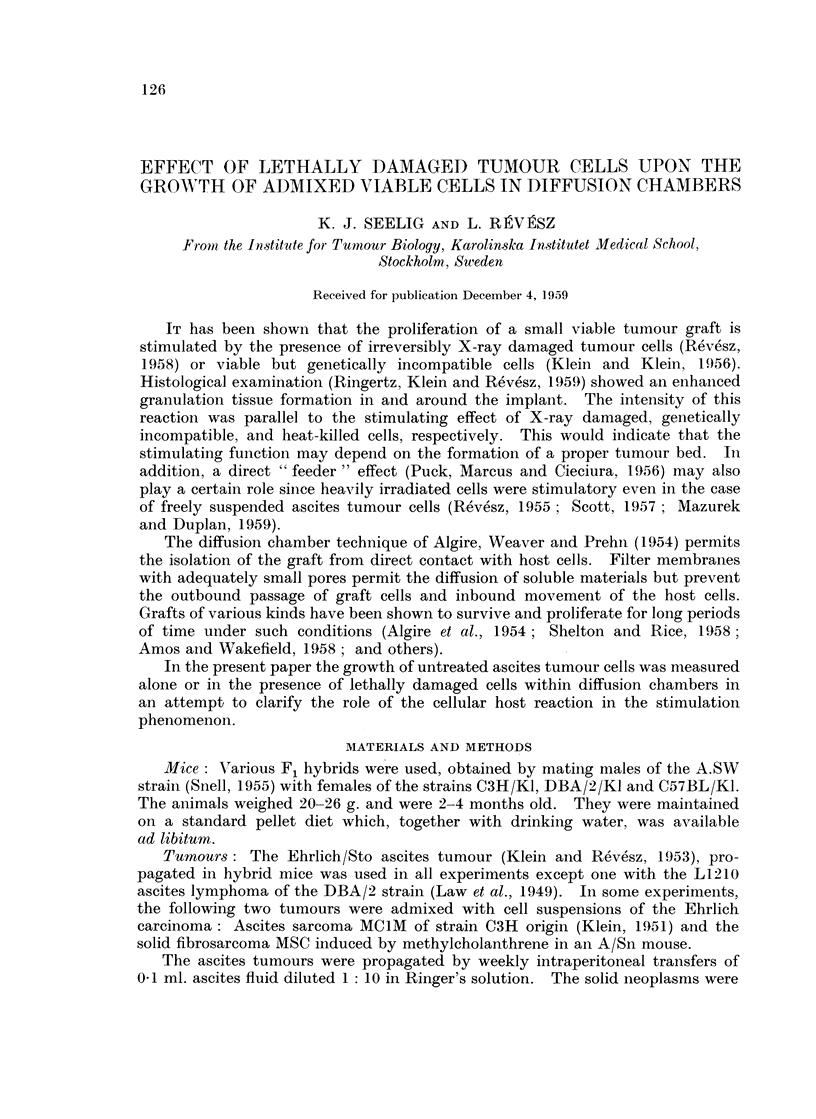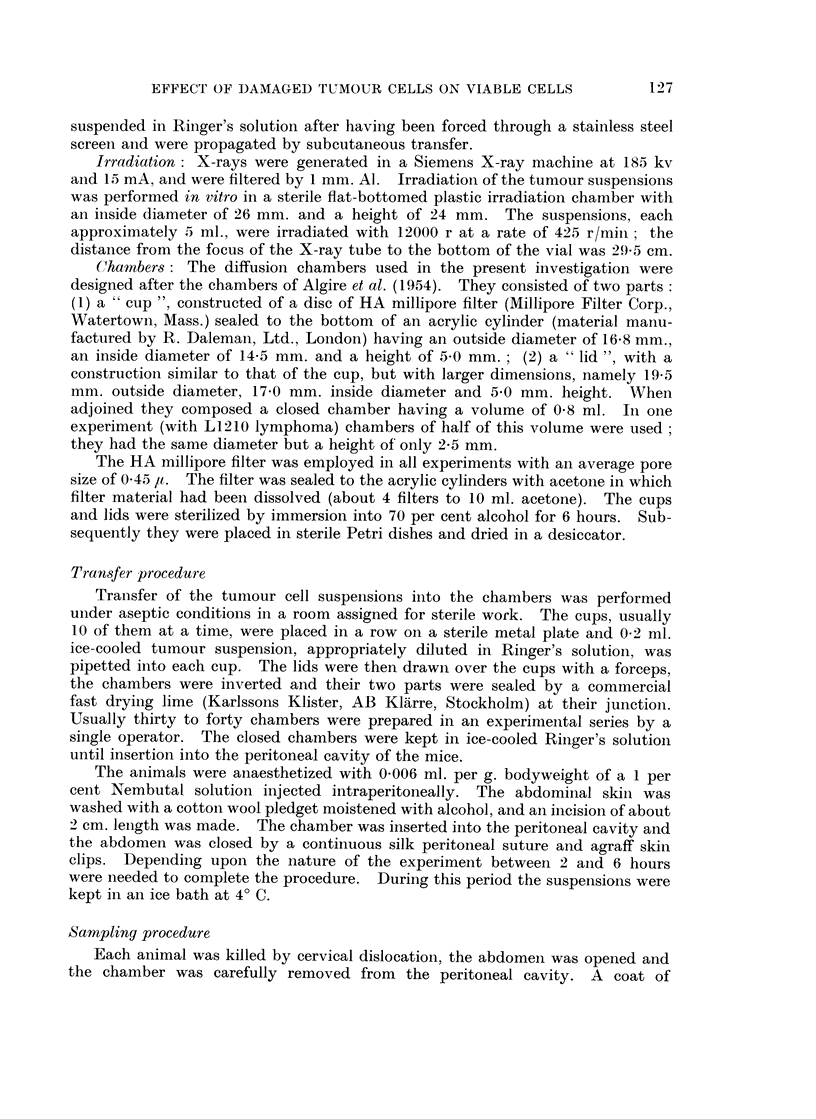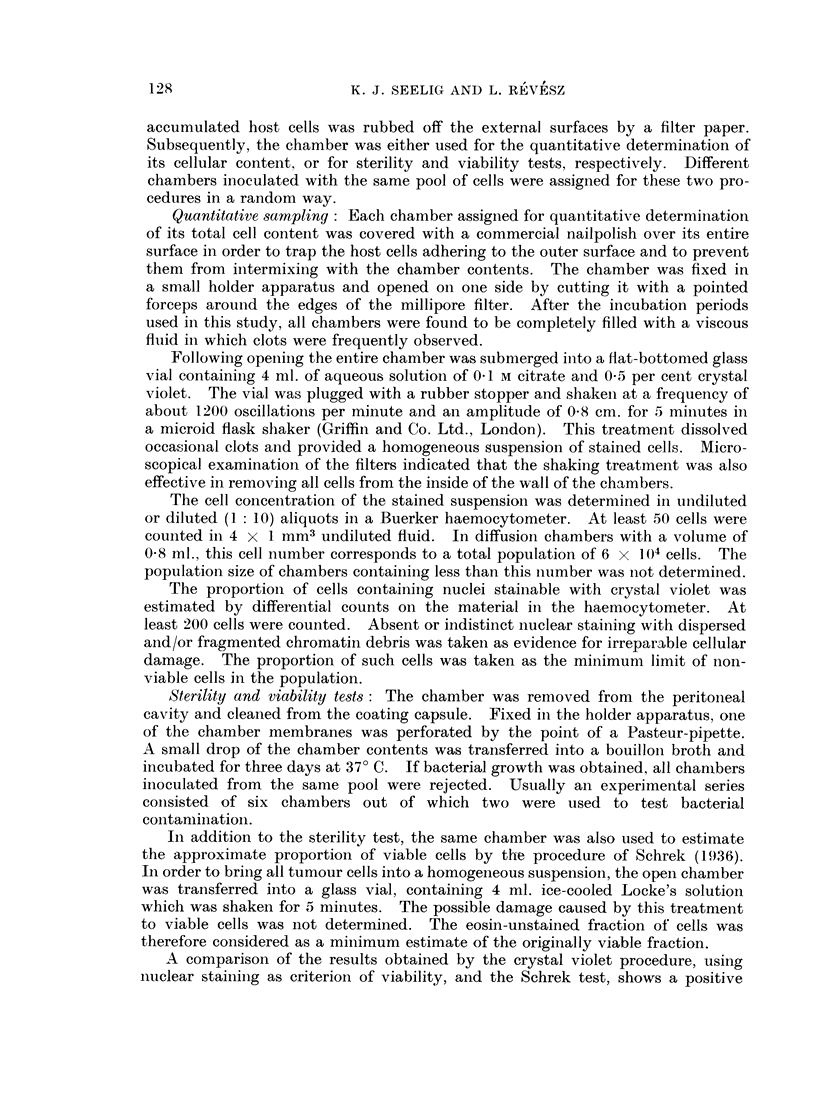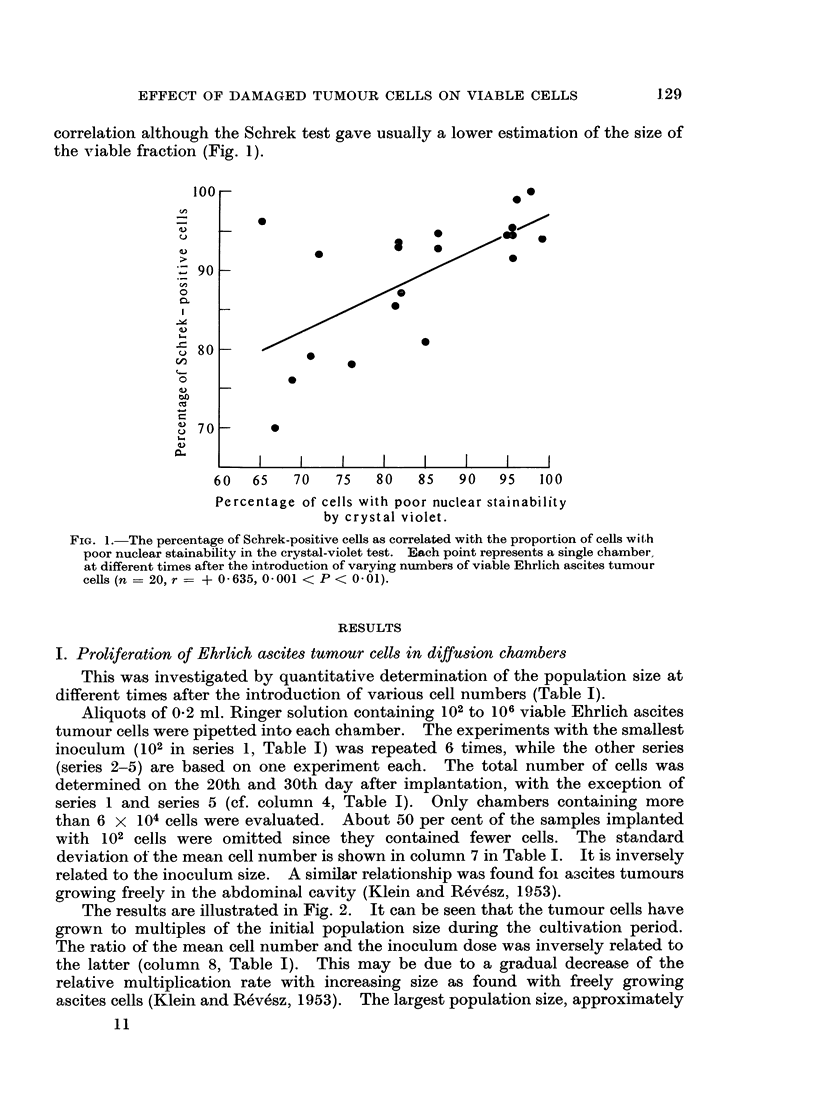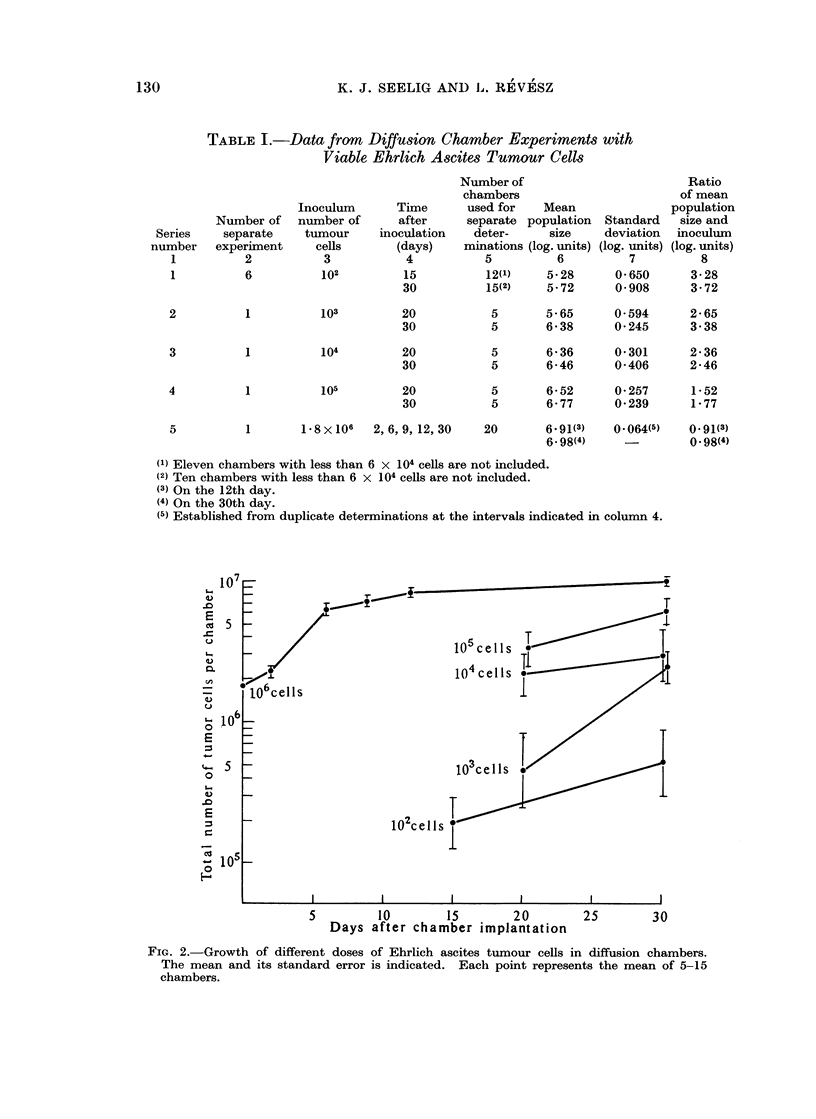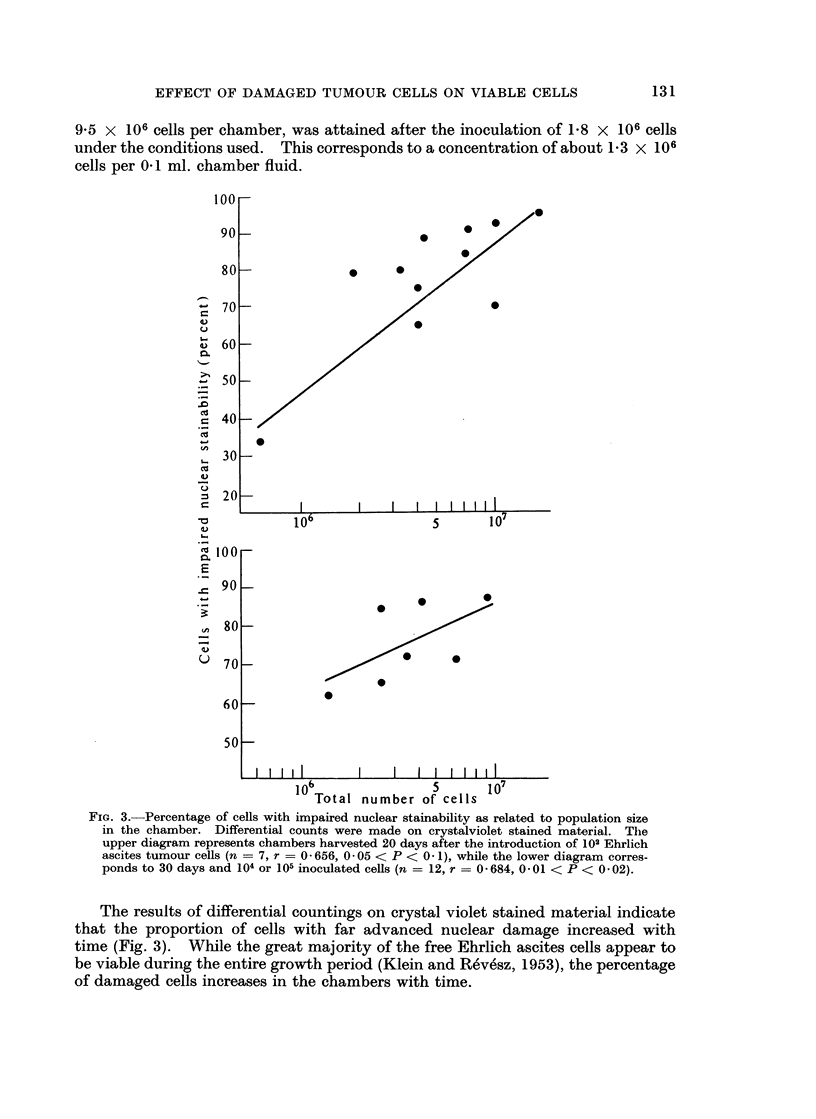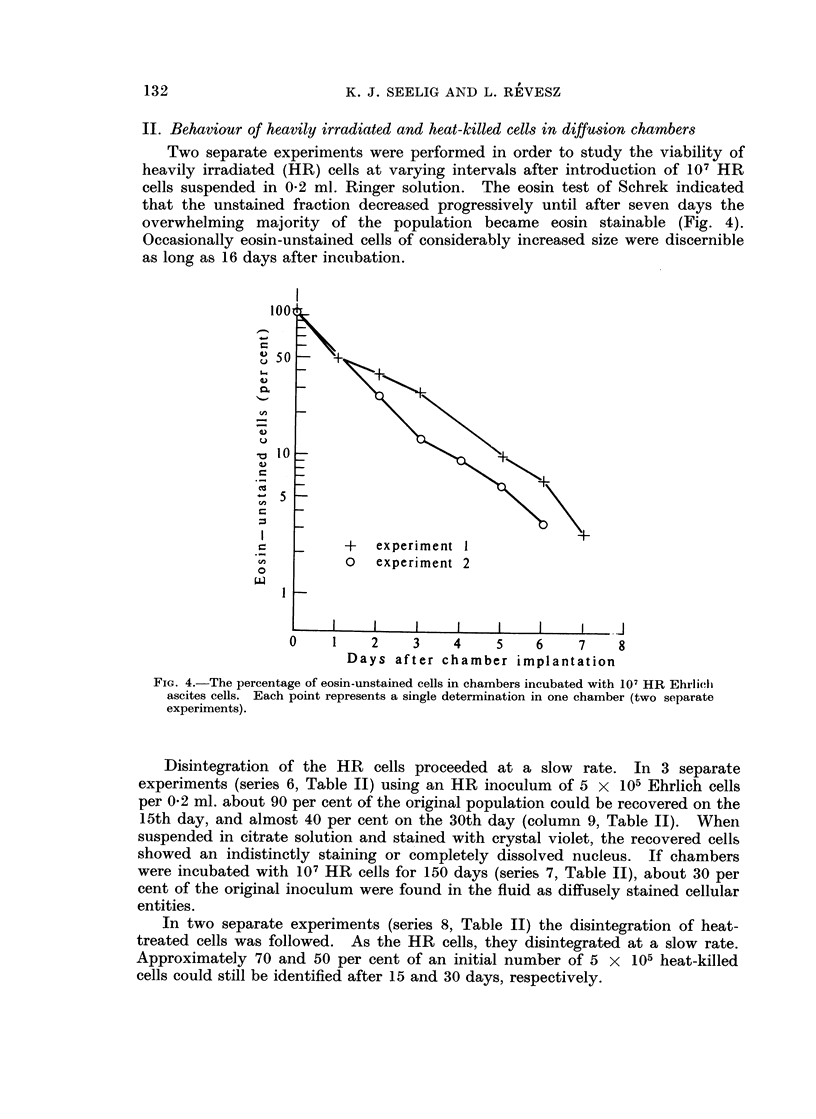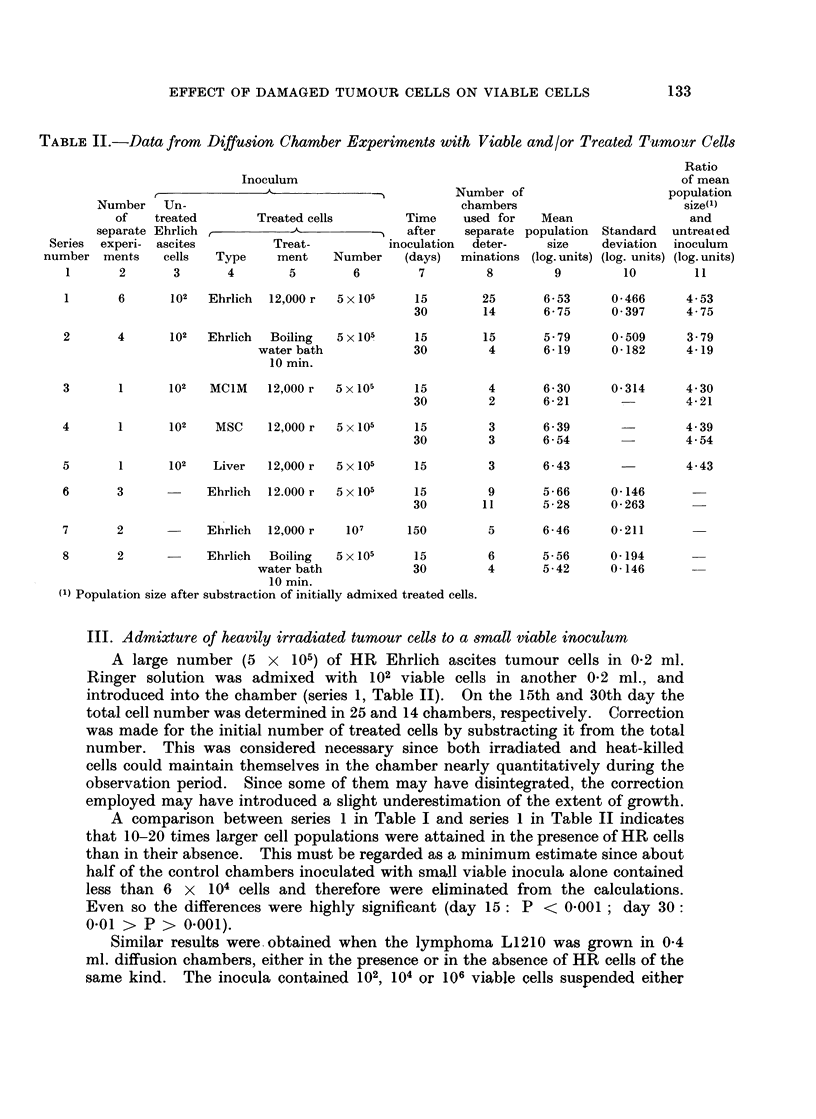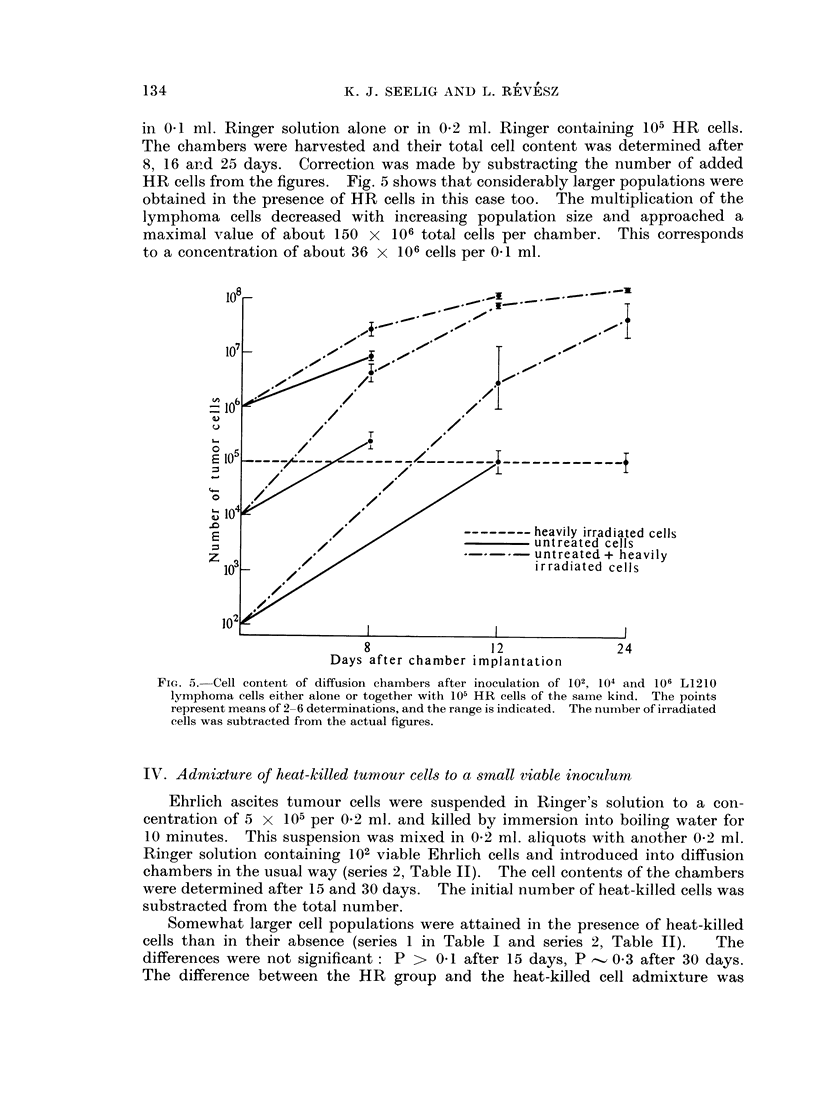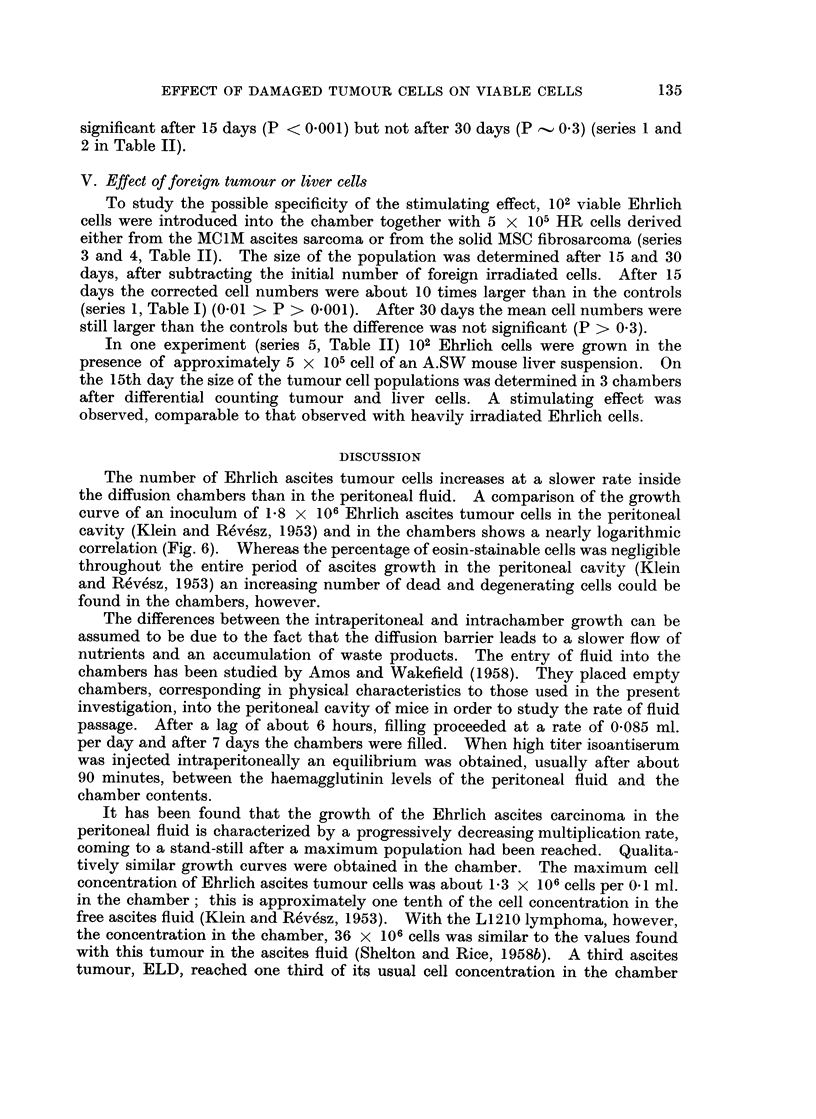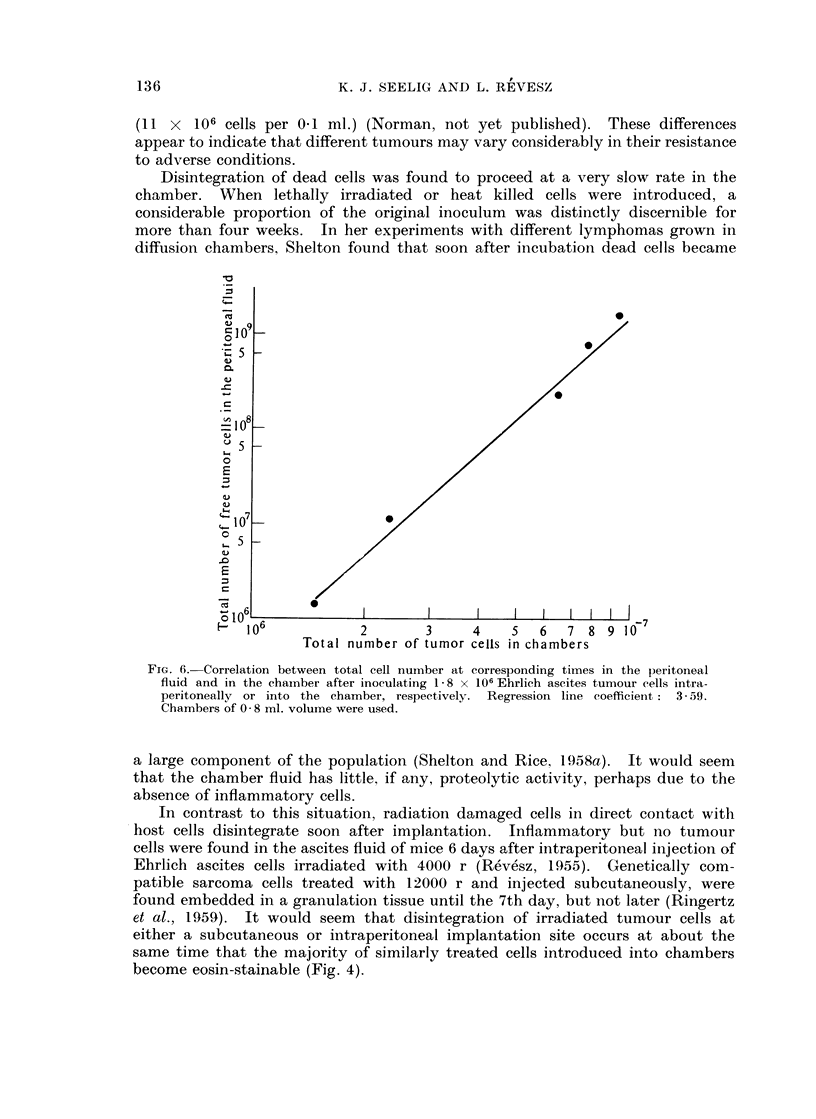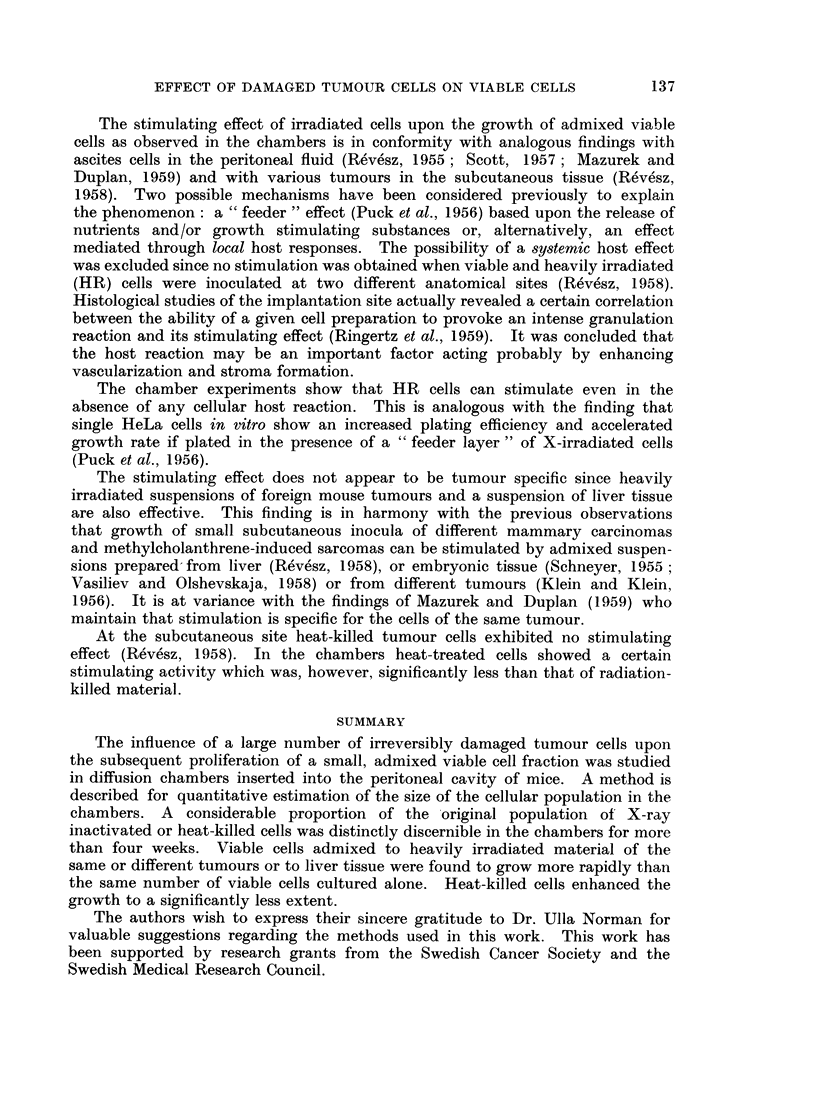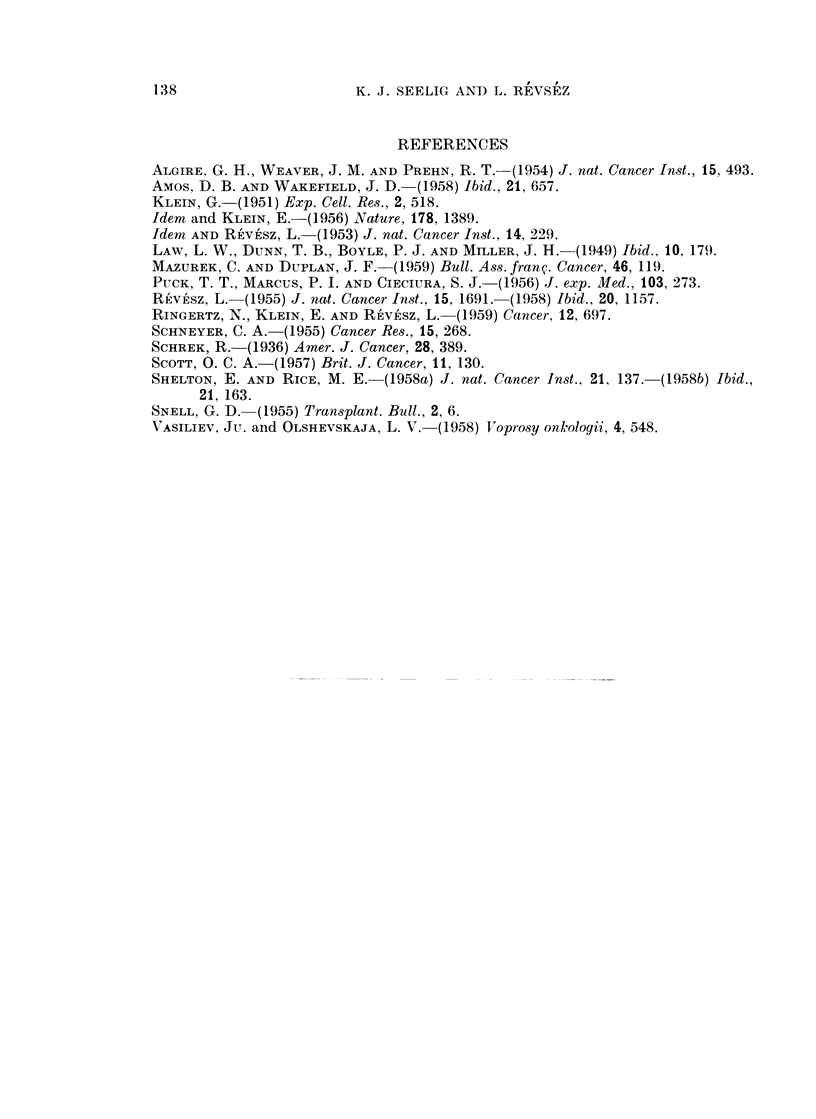# Effect of Lethally Damaged Tumour Cells upon the Growth of Admixed Viable Cells in Diffusion Chambers

**DOI:** 10.1038/bjc.1960.15

**Published:** 1960-03

**Authors:** K. J. Seelig, L. Révész


### EFFECT OF LETHALLY DAMAGED TUMOUR CELLS UPON THE GROWTH OF ADMIXED VIABLE CELLS IN DIFFUSION CHAMBERS

K. J. SEELIG AND L. RÉVÉSZ

*From the Institute for Tumour Biology, Karolinska Institutet Medical School, Stockholm, Sweden*

Received for publication December 4, 1959

It has been shown that the proliferation of a small viable tumour graft is stimulated by the presence of irreversibly X-ray damaged tumour cells (Révész, 1958) or viable but genetically incompatible cells (Klein and Klein, 1956). Histological examination (Ringertz, Klein and Révész, 1959) showed an enhanced granulation tissue formation in and around the implant. The intensity of this reaction was parallel to the stimulating effect of X-ray damaged, genetically incompatible, and heat-killed cells, respectively. This would indicate that the stimulating function may depend on the formation of a proper tumour bed. In addition, a direct "feeder" effect (Puck, Marcus and Cieciura, 1956) may also play a certain role since heavily irradiated cells were stimulatory even in the case of freely suspended ascites tumour cells (Révész, 1955; Scott, 1957; Mazurek and Duplan, 1959).

The diffusion chamber technique of Algire, Weaver and Prehn (1954) permits the isolation of the graft from direct contact with host cells. Filter membranes with adequately small pores permit the diffusion of soluble materials but prevent the outbound passage of graft cells and inbound movement of the host cells. Grafts of various kinds have been shown to survive and proliferate for long periods of time under such conditions (Algire *et al.*, 1954; Shelton and Rice, 1958; Amos and Wakefield, 1958; and others).

In the present paper the growth of untreated ascites tumour cells was measured alone or in the presence of lethally damaged cells within diffusion chambers in an attempt to clarify the role of the cellular host reaction in the stimulation phenomenon.

### MATERIALS AND METHODS

*Mice*: Various $F_1$ hybrids were used, obtained by mating males of the A.SW strain (Snell, 1955) with females of the strains C3H/Kl, DBA/2/Kl and C57BL/Kl. The animals weighed 20–26 g. and were 2–4 months old. They were maintained on a standard pellet diet which, together with drinking water, was available *ad libitum*.

*Tumours*: The Ehrlich/Sto ascites tumour (Klein and Révész, 1953), propagated in hybrid mice was used in all experiments except one with the L1210 ascites lymphoma of the DBA/2 strain (Law *et al.*, 1949). In some experiments, the following two tumours were admixed with cell suspensions of the Ehrlich carcinoma: Ascites sarcoma MC1M of strain C3H origin (Klein, 1951) and the solid fibrosarcoma MSC induced by methylcholanthrene in an A/Sn mouse.

The ascites tumours were propagated by weekly intraperitoneal transfers of 0·1 ml. ascites fluid diluted 1 : 10 in Ringer's solution. The solid neoplasms were suspended in Ringer's solution after having been forced through a stainless steel screen and were propagated by subcutaneous transfer.

*Irradiation* : X-rays were generated in a Siemens X-ray machine at 185 kv and 15 mA, and were filtered by 1 mm. Al. Irradiation of the tumour suspensions was performed *in vitro* in a sterile flat-bottomed plastic irradiation chamber with an inside diameter of 26 mm. and a height of 24 mm. The suspensions, each approximately 5 ml., were irradiated with 12000 r at a rate of 425 r/min ; the distance from the focus of the X-ray tube to the bottom of the vial was 29·5 cm.

*Chambers* : The diffusion chambers used in the present investigation were designed after the chambers of Algire *et al.* (1954). They consisted of two parts : (1) a " cup ", constructed of a disc of HA millipore filter (Millipore Filter Corp., Watertown, Mass.) sealed to the bottom of an acrylic cylinder (material manufactured by R. Daleman, Ltd., London) having an outside diameter of 16·8 mm., an inside diameter of 14·5 mm. and a height of 5·0 mm. ; (2) a " lid ", with a construction similar to that of the cup, but with larger dimensions, namely 19·5 mm. outside diameter, 17·0 mm. inside diameter and 5·0 mm. height. When adjoined they composed a closed chamber having a volume of 0·8 ml. In one experiment (with L1210 lymphoma) chambers of half of this volume were used ; they had the same diameter but a height of only 2·5 mm.

The HA millipore filter was employed in all experiments with an average pore size of 0·45 $\mu$. The filter was sealed to the acrylic cylinders with acetone in which filter material had been dissolved (about 4 filters to 10 ml. acetone). The cups and lids were sterilized by immersion into 70 per cent alcohol for 6 hours. Subsequently they were placed in sterile Petri dishes and dried in a desiccator.

*Transfer procedure*

Transfer of the tumour cell suspensions into the chambers was performed under aseptic conditions in a room assigned for sterile work. The cups, usually 10 of them at a time, were placed in a row on a sterile metal plate and 0·2 ml. ice-cooled tumour suspension, appropriately diluted in Ringer's solution, was pipetted into each cup. The lids were then drawn over the cups with a forceps, the chambers were inverted and their two parts were sealed by a commercial fast drying lime (Karlssons Klister, AB Klärre, Stockholm) at their junction. Usually thirty to forty chambers were prepared in an experimental series by a single operator. The closed chambers were kept in ice-cooled Ringer's solution until insertion into the peritoneal cavity of the mice.

The animals were anaesthetized with 0·006 ml. per g. bodyweight of a 1 per cent Nembutal solution injected intraperitoneally. The abdominal skin was washed with a cotton wool pledget moistened with alcohol, and an incision of about 2 cm. length was made. The chamber was inserted into the peritoneal cavity and the abdomen was closed by a continuous silk peritoneal suture and agraff skin clips. Depending upon the nature of the experiment between 2 and 6 hours were needed to complete the procedure. During this period the suspensions were kept in an ice bath at 4° C.

*Sampling procedure*

Each animal was killed by cervical dislocation, the abdomen was opened and the chamber was carefully removed from the peritoneal cavity. A coat of accumulated host cells was rubbed off the external surfaces by a filter paper. Subsequently, the chamber was either used for the quantitative determination of its cellular content, or for sterility and viability tests, respectively. Different chambers inoculated with the same pool of cells were assigned for these two procedures in a random way.

*Quantitative sampling* : Each chamber assigned for quantitative determination of its total cell content was covered with a commercial nailpolish over its entire surface in order to trap the host cells adhering to the outer surface and to prevent them from intermixing with the chamber contents. The chamber was fixed in a small holder apparatus and opened on one side by cutting it with a pointed forceps around the edges of the millipore filter. After the incubation periods used in this study, all chambers were found to be completely filled with a viscous fluid in which clots were frequently observed.

Following opening the entire chamber was submerged into a flat-bottomed glass vial containing 4 ml. of aqueous solution of 0·1 M citrate and 0·5 per cent crystal violet. The vial was plugged with a rubber stopper and shaken at a frequency of about 1200 oscillations per minute and an amplitude of 0·8 cm. for 5 minutes in a microid flask shaker (Griffin and Co. Ltd., London). This treatment dissolved occasional clots and provided a homogeneous suspension of stained cells. Microscopical examination of the filters indicated that the shaking treatment was also effective in removing all cells from the inside of the wall of the chambers.

The cell concentration of the stained suspension was determined in undiluted or diluted (1 : 10) aliquots in a Buerker haemocytometer. At least 50 cells were counted in $4 \times 1$ mm$^3$ undiluted fluid. In diffusion chambers with a volume of 0·8 ml., this cell number corresponds to a total population of $6 \times 10^4$ cells. The population size of chambers containing less than this number was not determined.

The proportion of cells containing nuclei stainable with crystal violet was estimated by differential counts on the material in the haemocytometer. At least 200 cells were counted. Absent or indistinct nuclear staining with dispersed and/or fragmented chromatin debris was taken as evidence for irreparable cellular damage. The proportion of such cells was taken as the minimum limit of non-viable cells in the population.

*Sterility and viability tests* : The chamber was removed from the peritoneal cavity and cleaned from the coating capsule. Fixed in the holder apparatus, one of the chamber membranes was perforated by the point of a Pasteur-pipette. A small drop of the chamber contents was transferred into a bouillon broth and incubated for three days at 37° C. If bacterial growth was obtained, all chambers inoculated from the same pool were rejected. Usually an experimental series consisted of six chambers out of which two were used to test bacterial contamination.

In addition to the sterility test, the same chamber was also used to estimate the approximate proportion of viable cells by the procedure of Schrek (1936). In order to bring all tumour cells into a homogeneous suspension, the open chamber was transferred into a glass vial, containing 4 ml. ice-cooled Locke's solution which was shaken for 5 minutes. The possible damage caused by this treatment to viable cells was not determined. The eosin-unstained fraction of cells was therefore considered as a minimum estimate of the originally viable fraction.

A comparison of the results obtained by the crystal violet procedure, using nuclear staining as criterion of viability, and the Schrek test, shows a positive correlation although the Schrek test gave usually a lower estimation of the size of the viable fraction (Fig. 1).

FIG. 1.—The percentage of Schrek-positive cells as correlated with the proportion of cells with poor nuclear stainability in the crystal-violet test. Each point represents a single chamber, at different times after the introduction of varying numbers of viable Ehrlich ascites tumour cells ($n = 20$, $r = + 0{\cdot}635$, $0{\cdot}001 < P < 0{\cdot}01$).

### RESULTS

### I. *Proliferation of Ehrlich ascites tumour cells in diffusion chambers*

This was investigated by quantitative determination of the population size at different times after the introduction of various cell numbers (Table I).

Aliquots of 0·2 ml. Ringer solution containing $10^2$ to $10^6$ viable Ehrlich ascites tumour cells were pipetted into each chamber. The experiments with the smallest inoculum ($10^2$ in series 1, Table I) was repeated 6 times, while the other series (series 2–5) are based on one experiment each. The total number of cells was determined on the 20th and 30th day after implantation, with the exception of series 1 and series 5 (cf. column 4, Table I). Only chambers containing more than $6 \times 10^4$ cells were evaluated. About 50 per cent of the samples implanted with $10^2$ cells were omitted since they contained fewer cells. The standard deviation of the mean cell number is shown in column 7 in Table I. It is inversely related to the inoculum size. A similar relationship was found foı ascites tumours growing freely in the abdominal cavity (Klein and Révész, 1953).

The results are illustrated in Fig. 2. It can be seen that the tumour cells have grown to multiples of the initial population size during the cultivation period. The ratio of the mean cell number and the inoculum dose was inversely related to the latter (column 8, Table I). This may be due to a gradual decrease of the relative multiplication rate with increasing size as found with freely growing ascites cells (Klein and Révész, 1953). The largest population size, approximately

TABLE I.—*Data from Diffusion Chamber Experiments with Viable Ehrlich Ascites Tumour Cells*

| Series number | Number of separate experiment | Inoculum number of tumour cells | Time after inoculation (days) | Number of chambers used for separate determinations | Mean population size (log. units) | Standard deviation (log. units) | Ratio of mean population size and inoculum (log. units) |
|---|---|---|---|---|---|---|---|
| 1 | 2 | 3 | 4 | 5 | 6 | 7 | 8 |
| 1 | 6 | $10^2$ | 15 | 12(1) | 5·28 | 0·650 | 3·28 |
| | | | 30 | 15(2) | 5·72 | 0·908 | 3·72 |
| 2 | 1 | $10^3$ | 20 | 5 | 5·65 | 0·594 | 2·65 |
| | | | 30 | 5 | 6·38 | 0·245 | 3·38 |
| 3 | 1 | $10^4$ | 20 | 5 | 6·36 | 0·301 | 2·36 |
| | | | 30 | 5 | 6·46 | 0·406 | 2·46 |
| 4 | 1 | $10^5$ | 20 | 5 | 6·52 | 0·257 | 1·52 |
| | | | 30 | 5 | 6·77 | 0·239 | 1·77 |
| 5 | 1 | $1·8 \times 10^6$ | 2, 6, 9, 12, 30 | 20 | 6·91(3) | 0·064(5) | 0·91(3) |
| | | | | | 6·98(4) | — | 0·98(4) |

(1) Eleven chambers with less than $6 \times 10^4$ cells are not included.
(2) Ten chambers with less than $6 \times 10^4$ cells are not included.
(3) On the 12th day.
(4) On the 30th day.
(5) Established from duplicate determinations at the intervals indicated in column 4.

FIG. 2.—Growth of different doses of Ehrlich ascites tumour cells in diffusion chambers. The mean and its standard error is indicated. Each point represents the mean of 5–15 chambers.

$9 \cdot 5 \times 10^6$ cells per chamber, was attained after the inoculation of $1 \cdot 8 \times 10^6$ cells under the conditions used. This corresponds to a concentration of about $1 \cdot 3 \times 10^6$ cells per 0·1 ml. chamber fluid.

FIG. 3.—Percentage of cells with impaired nuclear stainability as related to population size in the chamber. Differential counts were made on crystalviolet stained material. The upper diagram represents chambers harvested 20 days after the introduction of $10^2$ Ehrlich ascites tumour cells ($n = 7$, $r = 0 \cdot 656$, $0 \cdot 05 < P < 0 \cdot 1$), while the lower diagram corresponds to 30 days and $10^4$ or $10^5$ inoculated cells ($n = 12$, $r = 0 \cdot 684$, $0 \cdot 01 < P < 0 \cdot 02$).

The results of differential countings on crystal violet stained material indicate that the proportion of cells with far advanced nuclear damage increased with time (Fig. 3). While the great majority of the free Ehrlich ascites cells appear to be viable during the entire growth period (Klein and Révész, 1953), the percentage of damaged cells increases in the chambers with time.

II. *Behaviour of heavily irradiated and heat-killed cells in diffusion chambers*

Two separate experiments were performed in order to study the viability of heavily irradiated (HR) cells at varying intervals after introduction of $10^7$ HR cells suspended in 0·2 ml. Ringer solution. The eosin test of Schrek indicated that the unstained fraction decreased progressively until after seven days the overwhelming majority of the population became eosin stainable (Fig. 4). Occasionally eosin-unstained cells of considerably increased size were discernible as long as 16 days after incubation.

FIG. 4.—The percentage of eosin-unstained cells in chambers incubated with $10^7$ HR Ehrlich ascites cells. Each point represents a single determination in one chamber (two separate experiments).

Disintegration of the HR cells proceeded at a slow rate. In 3 separate experiments (series 6, Table II) using an HR inoculum of $5 \times 10^5$ Ehrlich cells per 0·2 ml. about 90 per cent of the original population could be recovered on the 15th day, and almost 40 per cent on the 30th day (column 9, Table II). When suspended in citrate solution and stained with crystal violet, the recovered cells showed an indistinctly staining or completely dissolved nucleus. If chambers were incubated with $10^7$ HR cells for 150 days (series 7, Table II), about 30 per cent of the original inoculum were found in the fluid as diffusely stained cellular entities.

In two separate experiments (series 8, Table II) the disintegration of heat-treated cells was followed. As the HR cells, they disintegrated at a slow rate. Approximately 70 and 50 per cent of an initial number of $5 \times 10^5$ heat-killed cells could still be identified after 15 and 30 days, respectively.

TABLE II.—*Data from Diffusion Chamber Experiments with Viable and/or Treated Tumour Cells*

| Series number | Number of separate experiments | Inoculum: Untreated Ehrlich ascites cells | Treated cells: Type | Treated cells: Treatment | Treated cells: Number | Time after inoculation (days) | Number of chambers used for separate determinations | Mean population size (log. units) | Standard deviation (log. units) | Ratio of mean population size[1] and untreated inoculum (log. units) |
|---|---|---|---|---|---|---|---|---|---|---|
| 1 | 2 | 3 | 4 | 5 | 6 | 7 | 8 | 9 | 10 | 11 |
| 1 | 6 | $10^2$ | Ehrlich | 12,000 r | $5 \times 10^5$ | 15 | 25 | 6·53 | 0·466 | 4·53 |
| | | | | | | 30 | 14 | 6·75 | 0·397 | 4·75 |
| 2 | 4 | $10^2$ | Ehrlich | Boiling water bath 10 min. | $5 \times 10^5$ | 15 | 15 | 5·79 | 0·509 | 3·79 |
| | | | | | | 30 | 4 | 6·19 | 0·182 | 4·19 |
| 3 | 1 | $10^2$ | MC1M | 12,000 r | $5 \times 10^5$ | 15 | 4 | 6·30 | 0·314 | 4·30 |
| | | | | | | 30 | 2 | 6·21 | — | 4·21 |
| 4 | 1 | $10^2$ | MSC | 12,000 r | $5 \times 10^5$ | 15 | 3 | 6·39 | — | 4·39 |
| | | | | | | 30 | 3 | 6·54 | — | 4·54 |
| 5 | 1 | $10^2$ | Liver | 12,000 r | $5 \times 10^5$ | 15 | 3 | 6·43 | — | 4·43 |
| 6 | 3 | — | Ehrlich | 12.000 r | $5 \times 10^5$ | 15 | 9 | 5·66 | 0·146 | — |
| | | | | | | 30 | 11 | 5·28 | 0·263 | — |
| 7 | 2 | — | Ehrlich | 12,000 r | $10^7$ | 150 | 5 | 6·46 | 0·211 | — |
| 8 | 2 | — | Ehrlich | Boiling water bath 10 min. | $5 \times 10^5$ | 15 | 6 | 5·56 | 0·194 | — |
| | | | | | | 30 | 4 | 5·42 | 0·146 | — |

(1) Population size after substraction of initially admixed treated cells.

### III. *Admixture of heavily irradiated tumour cells to a small viable inoculum*

A large number ($5 \times 10^5$) of HR Ehrlich ascites tumour cells in 0·2 ml. Ringer solution was admixed with $10^2$ viable cells in another 0·2 ml., and introduced into the chamber (series 1, Table II). On the 15th and 30th day the total cell number was determined in 25 and 14 chambers, respectively. Correction was made for the initial number of treated cells by substracting it from the total number. This was considered necessary since both irradiated and heat-killed cells could maintain themselves in the chamber nearly quantitatively during the observation period. Since some of them may have disintegrated, the correction employed may have introduced a slight underestimation of the extent of growth.

A comparison between series 1 in Table I and series 1 in Table II indicates that 10–20 times larger cell populations were attained in the presence of HR cells than in their absence. This must be regarded as a minimum estimate since about half of the control chambers inoculated with small viable inocula alone contained less than $6 \times 10^4$ cells and therefore were eliminated from the calculations. Even so the differences were highly significant (day 15 : $P < 0{\cdot}001$ ; day 30 : $0{\cdot}01 > P > 0{\cdot}001$).

Similar results were obtained when the lymphoma L1210 was grown in 0·4 ml. diffusion chambers, either in the presence or in the absence of HR cells of the same kind. The inocula contained $10^2$, $10^4$ or $10^6$ viable cells suspended either in 0·1 ml. Ringer solution alone or in 0·2 ml. Ringer containing $10^5$ HR cells. The chambers were harvested and their total cell content was determined after 8, 16 and 25 days. Correction was made by substracting the number of added HR cells from the figures. Fig. 5 shows that considerably larger populations were obtained in the presence of HR cells in this case too. The multiplication of the lymphoma cells decreased with increasing population size and approached a maximal value of about 150 × $10^6$ total cells per chamber. This corresponds to a concentration of about 36 × $10^6$ cells per 0·1 ml.

FIG. 5.—Cell content of diffusion chambers after inoculation of $10^2$, $10^4$ and $10^6$ L1210 lymphoma cells either alone or together with $10^5$ HR cells of the same kind. The points represent means of 2–6 determinations, and the range is indicated. The number of irradiated cells was subtracted from the actual figures.

IV. *Admixture of heat-killed tumour cells to a small viable inoculum*

Ehrlich ascites tumour cells were suspended in Ringer's solution to a concentration of 5 × $10^5$ per 0·2 ml. and killed by immersion into boiling water for 10 minutes. This suspension was mixed in 0·2 ml. aliquots with another 0·2 ml. Ringer solution containing $10^2$ viable Ehrlich cells and introduced into diffusion chambers in the usual way (series 2, Table II). The cell contents of the chambers were determined after 15 and 30 days. The initial number of heat-killed cells was substracted from the total number.

Somewhat larger cell populations were attained in the presence of heat-killed cells than in their absence (series 1 in Table I and series 2, Table II). The differences were not significant : $P > 0{\cdot}1$ after 15 days, $P \sim 0{\cdot}3$ after 30 days. The difference between the HR group and the heat-killed cell admixture was significant after 15 days ($P < 0{\cdot}001$) but not after 30 days ($P \sim 0{\cdot}3$) (series 1 and 2 in Table II).

V. *Effect of foreign tumour or liver cells*

To study the possible specificity of the stimulating effect, $10^2$ viable Ehrlich cells were introduced into the chamber together with $5 \times 10^5$ HR cells derived either from the MC1M ascites sarcoma or from the solid MSC fibrosarcoma (series 3 and 4, Table II). The size of the population was determined after 15 and 30 days, after subtracting the initial number of foreign irradiated cells. After 15 days the corrected cell numbers were about 10 times larger than in the controls (series 1, Table I) ($0{\cdot}01 > P > 0{\cdot}001$). After 30 days the mean cell numbers were still larger than the controls but the difference was not significant ($P > 0{\cdot}3$).

In one experiment (series 5, Table II) $10^2$ Ehrlich cells were grown in the presence of approximately $5 \times 10^5$ cell of an A.SW mouse liver suspension. On the 15th day the size of the tumour cell populations was determined in 3 chambers after differential counting tumour and liver cells. A stimulating effect was observed, comparable to that observed with heavily irradiated Ehrlich cells.

### DISCUSSION

The number of Ehrlich ascites tumour cells increases at a slower rate inside the diffusion chambers than in the peritoneal fluid. A comparison of the growth curve of an inoculum of $1{\cdot}8 \times 10^6$ Ehrlich ascites tumour cells in the peritoneal cavity (Klein and Révész, 1953) and in the chambers shows a nearly logarithmic correlation (Fig. 6). Whereas the percentage of eosin-stainable cells was negligible throughout the entire period of ascites growth in the peritoneal cavity (Klein and Révész, 1953) an increasing number of dead and degenerating cells could be found in the chambers, however.

The differences between the intraperitoneal and intrachamber growth can be assumed to be due to the fact that the diffusion barrier leads to a slower flow of nutrients and an accumulation of waste products. The entry of fluid into the chambers has been studied by Amos and Wakefield (1958). They placed empty chambers, corresponding in physical characteristics to those used in the present investigation, into the peritoneal cavity of mice in order to study the rate of fluid passage. After a lag of about 6 hours, filling proceeded at a rate of 0·085 ml. per day and after 7 days the chambers were filled. When high titer isoantiserum was injected intraperitoneally an equilibrium was obtained, usually after about 90 minutes, between the haemagglutinin levels of the peritoneal fluid and the chamber contents.

It has been found that the growth of the Ehrlich ascites carcinoma in the peritoneal fluid is characterized by a progressively decreasing multiplication rate, coming to a stand-still after a maximum population had been reached. Qualitatively similar growth curves were obtained in the chamber. The maximum cell concentration of Ehrlich ascites tumour cells was about $1{\cdot}3 \times 10^6$ cells per 0·1 ml. in the chamber ; this is approximately one tenth of the cell concentration in the free ascites fluid (Klein and Révész, 1953). With the L1210 lymphoma, however, the concentration in the chamber, $36 \times 10^6$ cells was similar to the values found with this tumour in the ascites fluid (Shelton and Rice, 1958*b*). A third ascites tumour, ELD, reached one third of its usual cell concentration in the chamber

(11 × $10^6$ cells per 0·1 ml.) (Norman, not yet published). These differences appear to indicate that different tumours may vary considerably in their resistance to adverse conditions.

Disintegration of dead cells was found to proceed at a very slow rate in the chamber. When lethally irradiated or heat killed cells were introduced, a considerable proportion of the original inoculum was distinctly discernible for more than four weeks. In her experiments with different lymphomas grown in diffusion chambers, Shelton found that soon after incubation dead cells became

Fig. 6.—Correlation between total cell number at corresponding times in the peritoneal fluid and in the chamber after inoculating 1·8 × $10^6$ Ehrlich ascites tumour cells intraperitoneally or into the chamber, respectively. Regression line coefficient : 3·59. Chambers of 0·8 ml. volume were used.

a large component of the population (Shelton and Rice, 1958*a*). It would seem that the chamber fluid has little, if any, proteolytic activity, perhaps due to the absence of inflammatory cells.

In contrast to this situation, radiation damaged cells in direct contact with host cells disintegrate soon after implantation. Inflammatory but no tumour cells were found in the ascites fluid of mice 6 days after intraperitoneal injection of Ehrlich ascites cells irradiated with 4000 r (Révész, 1955). Genetically compatible sarcoma cells treated with 12000 r and injected subcutaneously, were found embedded in a granulation tissue until the 7th day, but not later (Ringertz *et al.*, 1959). It would seem that disintegration of irradiated tumour cells at either a subcutaneous or intraperitoneal implantation site occurs at about the same time that the majority of similarly treated cells introduced into chambers become eosin-stainable (Fig. 4).

The stimulating effect of irradiated cells upon the growth of admixed viable cells as observed in the chambers is in conformity with analogous findings with ascites cells in the peritoneal fluid (Révész, 1955 ; Scott, 1957 ; Mazurek and Duplan, 1959) and with various tumours in the subcutaneous tissue (Révész, 1958). Two possible mechanisms have been considered previously to explain the phenomenon : a " feeder " effect (Puck *et al.*, 1956) based upon the release of nutrients and/or growth stimulating substances or, alternatively, an effect mediated through *local* host responses. The possibility of a *systemic* host effect was excluded since no stimulation was obtained when viable and heavily irradiated (HR) cells were inoculated at two different anatomical sites (Révész, 1958). Histological studies of the implantation site actually revealed a certain correlation between the ability of a given cell preparation to provoke an intense granulation reaction and its stimulating effect (Ringertz *et al.*, 1959). It was concluded that the host reaction may be an important factor acting probably by enhancing vascularization and stroma formation.

The chamber experiments show that HR cells can stimulate even in the absence of any cellular host reaction. This is analogous with the finding that single HeLa cells *in vitro* show an increased plating efficiency and accelerated growth rate if plated in the presence of a " feeder layer " of X-irradiated cells (Puck *et al.*, 1956).

The stimulating effect does not appear to be tumour specific since heavily irradiated suspensions of foreign mouse tumours and a suspension of liver tissue are also effective. This finding is in harmony with the previous observations that growth of small subcutaneous inocula of different mammary carcinomas and methylcholanthrene-induced sarcomas can be stimulated by admixed suspensions prepared from liver (Révész, 1958), or embryonic tissue (Schneyer, 1955 ; Vasiliev and Olshevskaja, 1958) or from different tumours (Klein and Klein, 1956). It is at variance with the findings of Mazurek and Duplan (1959) who maintain that stimulation is specific for the cells of the same tumour.

At the subcutaneous site heat-killed tumour cells exhibited no stimulating effect (Révész, 1958). In the chambers heat-treated cells showed a certain stimulating activity which was, however, significantly less than that of radiation-killed material.

### SUMMARY

The influence of a large number of irreversibly damaged tumour cells upon the subsequent proliferation of a small, admixed viable cell fraction was studied in diffusion chambers inserted into the peritoneal cavity of mice. A method is described for quantitative estimation of the size of the cellular population in the chambers. A considerable proportion of the original population of X-ray inactivated or heat-killed cells was distinctly discernible in the chambers for more than four weeks. Viable cells admixed to heavily irradiated material of the same or different tumours or to liver tissue were found to grow more rapidly than the same number of viable cells cultured alone. Heat-killed cells enhanced the growth to a significantly less extent.

The authors wish to express their sincere gratitude to Dr. Ulla Norman for valuable suggestions regarding the methods used in this work. This work has been supported by research grants from the Swedish Cancer Society and the Swedish Medical Research Council.

### REFERENCES

ALGIRE, G. H., WEAVER, J. M. AND PREHN, R. T.—(1954) *J. nat. Cancer Inst.*, **15**, 493.
AMOS, D. B. AND WAKEFIELD, J. D.—(1958) *Ibid.*, **21**, 657.
KLEIN, G.—(1951) *Exp. Cell. Res.*, **2**, 518.
*Idem* and KLEIN, E.—(1956) *Nature*, **178**, 1389.
*Idem* AND RÉVÉSZ, L.—(1953) *J. nat. Cancer Inst.*, **14**, 229.
LAW, L. W., DUNN, T. B., BOYLE, P. J. AND MILLER, J. H.—(1949) *Ibid.*, **10**, 179.
MAZUREK, C. AND DUPLAN, J. F.—(1959) *Bull. Ass. franç. Cancer*, **46**, 119.
PUCK, T. T., MARCUS, P. I. AND CIECIURA, S. J.—(1956) *J. exp. Med.*, **103**, 273.
RÉVÉSZ, L.—(1955) *J. nat. Cancer Inst.*, **15**, 1691.—(1958) *Ibid.*, **20**, 1157.
RINGERTZ, N., KLEIN, E. AND RÉVÉSZ, L.—(1959) *Cancer*, **12**, 697.
SCHNEYER, C. A.—(1955) *Cancer Res.*, **15**, 268.
SCHREK, R.—(1936) *Amer. J. Cancer*, **28**, 389.
SCOTT, O. C. A.—(1957) *Brit. J. Cancer*, **11**, 130.
SHELTON, E. AND RICE, M. E.—(1958*a*) *J. nat. Cancer Inst.*, **21**, 137.—(1958*b*) *Ibid.*, **21**, 163.
SNELL, G. D.—(1955) *Transplant. Bull.*, **2**, 6.
VASILIEV, JU. and OLSHEVSKAJA, L. V.—(1958) *Voprosy onkologii*, **4**, 548.